# The Mediating Role of Gaming Disorder in the Effect of Narcissism on Happiness in Children

**DOI:** 10.3390/ijerph18137137

**Published:** 2021-07-03

**Authors:** Orhan Çevik, Orhan Koçak, Mustafa Z. Younis, Elif Çevik

**Affiliations:** 1 Department of Social Work, Faculty of Health Sciences, Istanbul University—Cerrahpasa, Istanbul 34500, Turkey; oorhan.cevikk@gmail.com; 2 College of Health Sciences, Jackson State University, Jackson, MS 39217, USA; younis99@gmail.com; 3 Social Policy and Social Work, Institution of Social Sciences, Istanbul University, Istanbul 34500, Turkey; eliffcevikk@gmail.com

**Keywords:** compensation theory, gaming disorder, happiness, narcissism

## Abstract

We aimed to determine the relationship between gaming disorder, narcissism, and happiness levels of children between the ages of 9 and 15. This study was based on the compensation theory. The sample consists of 461 boys who continue their education in public schools in Istanbul. In the study, a mixed research design, which nests qualitative data into quantitative, was used. In addition to the scales and sociodemographic form, the Draw-a-Person test was also used to better understand children’s inner world. According to the findings, there is a significant relationship between gaming disorder and narcissism and happiness levels in children. Accordingly, as narcissism increases in children, the gaming disorder level increases, and happiness decreases. We also found a mediation effect in the impact of narcissism on happiness through gaming disorder. According to the results, we think that the problem is not caused by the individual but by society. For a solution, we recommend making more macro-level social work interventions within the framework of system theory instead of the current medical model in combating gaming disorder.

## 1. Introduction

Digital game playing, which has spread rapidly worldwide, has increased enormously with the COVID-19 pandemic [1]. WHO’s encouragement to play digital games during the stay-at-home process has also affected this increase [2]. In the first months of COVID-19, 30% of internet users in the United States and the United Kingdom played digital games more, 31% played the same amount, 7% played less, and 2% started playing for the first time [3]. According to the latest research, the number of people actively playing digital games reached 2.69 billion globally in 2020. This number was estimated to be 2.81 billion in 2021 and 3.07 billion in 2023 [4]. It was estimated that 56% of the people playing digital games are from the Y generation between the ages of 23 and 38 in 2020 [5]. Global game e-commerce increased by 23% from the previous year with the effect of the pandemic, reaching $135.8 billion in 2020, and this number is expected to reach $200 million in 2023 [6]. Throughout the world, 39% of those who played digital games played or downloaded a free video game in a month, and 18% watched a live game broadcast [3]. Among the most downloaded mobile game in the world was Among Us!, and the highest revenue-generating game was Pokémon GO [7]. League of Legends (LoL) was the most viewed game on Twitch, a platform where live video games are watched, in November 2020 [8,9].

With the rising rate of digital gaming, gaming disorder, a kind of behavioral addiction, has also been increasing. A study indicated that prior to the pandemic, numerous studies discovered a prevalence of gaming disorder ranging from 0.7 to 27.5% [10]. Another study conducted during the pandemic process has shown that the prevalence of gaming disorder has increased more than before [11].

Digital games, which are mostly played to spend time, relax, reduce stress, keep the mind active, dive into a different world, and socialize with friends or family members, cause many biological and psychological disorders besides behavioral addiction [12]. They cause more severe problems in children whose personality is not fully established and whose biological development is not fully completed [13].

Narcissism, one of these disorders, is a phenomenon increasing together with gaming disorder. Some studies showed that there was a 30% increase in the scores obtained from 16,275 university students who completed the narcissistic personality inventory in the years between 1979 and 2006. A study conducted by the U.S. National Institutes of Health in 2008 found that 6.2% of 35,000 participants showed narcissistic personality disorder at some time in their lives [14]. In addition, it was found that narcissism has increased much faster with the spread of social media [15,16,17,18].

The other disorder related to gaming disorder is unhappiness. According to the 2020 World Happiness Report, happiness has globally decreased, and anger, sadness, and anxiety have increased since 2014. Even in countries such as the Netherlands, the United States, and France, where happiness levels are high [19], recent riots, large-scale protests, and terrorist acts are a concrete indicator of the anger expression of general unhappiness in the world [20,21].

The current study, which was founded on Alfred Adler’s compensation theory [22], reveals the associations between narcissism, gaming disorder, and happiness. According to the findings, as children’s narcissism rises, so does their gaming disorder, while their happiness falls. We also found that gaming disorder mediates narcissism and happiness while playing LoL moderates narcissism and gaming disorder.

### 1.1. Gaming Disorder

Gaming disorder, also known as digital game addiction, video game addiction, and internet gaming disorder, among other names, is defined as a condition in which individuals cannot control their desire to play digital games and individuals’ emotions, thoughts, behaviors, and daily life change and deteriorate due to playing digital games [23]. The gaming disorder concept was accepted as a mental disorder among behavioral addictions in ICD-11 [24]. However, it, named internet gaming disorder by the American Psychiatric Association, has not yet been defined as a mental disorder in DSM-5, arguing that new studies are needed [25]. Furthermore, although the internet gaming disorder concept includes offline games in its definition, some researchers stated that the word “internet” limits the concept to online games only [26]. Since it is a more comprehensive concept, we also preferred to use the concept of gaming disorder instead of internet gaming disorder in this study.

### 1.2. Narcissism

Narcissism, which is defined in the dictionary as the admiration and devotion of one’s own bodily and spiritual self [27], was considered as a one-dimensional pathology in the early periods when it was the subject of scientific research [28]. However, it was suggested that narcissism has a healthy dimension in addition to its pathological, in other words, unhealthy, dimension [29].

Narcissism is classified as pathological and normal in many sources [30]. While pathological narcissism is considered a narcissistic personality disorder, normal narcissism is seen as a narcissistic personality trait [31]. This situation causes narcissism to be confused with self-confidence and self-esteem personality traits or to perceive normal narcissism as the beginning of undiagnosed pathological narcissism [32]. So, it would be healthier to define the level of self-love that every individual should have to establish healthy relationships with both themselves and their environment as healthy narcissism and to define the level of self-love that causes unhealthy relationships with both themselves and their environment as unhealthy narcissism [33].

### 1.3. Happiness

Happiness, defined as the state of being pleased of reaching all aspirations completely and continuously [27], has been discussed since ancient times, but there is still no consensus on what it exactly is. While some sources in the modern scientific literature explain happiness with basic concepts such as pleasure and desire, some have tried to explain it with very close concepts such as wellness, well-being, subjective well-being, psychological well-being, life satisfaction, quality of life, and positive affect [34].

Some classical theorists interpreted happiness as equated with momentary pleasure. According to them, every action that includes pleasure makes the individual happy [35], [36]. However, according to positive psychology theorists, happiness is a phenomenon that increases the individual’s quality of life and provides long-term satisfaction. To achieve this satisfaction, the individual must work hard [37]. According to the flow theory, it also becomes possible for individuals with high life satisfaction to give short-term pleasure to long-term happiness. However, these short-term pleasure objects make individuals with low life satisfaction dependent on themselves until they reach life satisfaction [38,39,40].

### 1.4. Gaming Disorder, Narcissism, and Happiness and the Present Research

When the literature was examined, no specific studies were found investigating the relationship between gaming disorder, narcissism, and happiness. However, it was seen that some studies found positive associations between healthy narcissism and happiness [41,42,43,44,45,46,47,48,49], and some found a negative relationship between unhealthy narcissism and happiness [41,43,44,50,51]. However, some studies found a positive relationship between unhealthy narcissism and happiness [48,52]. Many studies found a positive relationship between gaming disorder and narcissism [53]. The studies determined a positive relationship between technology addiction-related gaming disorder and narcissism [15,16,17,18,53]. Moreover, some studies found a negative relationship between gaming disorder and happiness [54,55,56,57,58,59,60,61,62,63,64,65,66]. In contrast, some found a positive relationship between gaming disorder and happiness levels [67]. In a nutshell, most studies found a positive relationship between gaming disorder and narcissism and a negative association between gaming disorder and happiness. A positive relationship was mostly found between narcissism and happiness.

In this study, we tried to explain the relationship between these three concepts in the light of compensation theory supporting psychosocial development theory. According to the compensation theory, the individual tries to get rid of the inferiority complex and compensate for the level of narcissism through games [22]. When this compensation occurs in traditional games, the individual reaches a healthy narcissism level and becomes happy, whereas, in digital games, the individual reaches unhealthy narcissism and becomes unhappy. No scales were found in the literature that measures narcissism with its healthy and unhealthy dimensions. Some scales are based solely on pathological narcissism [42,68], whereas some measure only normal narcissism [69,70]. However, while pathological narcissism scales consist of items containing only unhealthy narcissism expressions, there are questions measuring both healthy and unhealthy narcissism in normal narcissism scales, such as the one we used in this study. It is not clear exactly which type of narcissism these scales measure. Therefore, as the scales used in studies on narcissism are thought to be problematic due to unclear subdimensions [71], an attempt was made to validate the hypotheses by looking at the relationship between happiness and gaming disorder to understand the type of narcissism compensated for. With the assumption that gaming disorder has a mediating role in the relation between narcissism and happiness, and the Draw-a-Person test, the associations between these three variables were made more understandable.

### 1.5. Theoretical Background and Hypotheses

According to the compensation theory developed by Alfred Adler, individuals who are in inferiority complex and fail to achieve the expected try to compensate for their level of narcissism by turning to different areas where they can get approval to get rid of their inferiority complexes. If individuals can get the approval of the environment differently, compensation will occur, and they will reach the desired level of narcissism in a healthy way. The individuals who cannot find a space to show themselves and receive approval despite the effort made try to obtain the praise and approval they need in different ways and reach unhealthy narcissism [22]. Individuals may be unhappy because they receive the approval of a different environment or object, not the environment they belong to. However, this consent is not respected in real life and is even further excluded. The more they are excluded, the more they attach to the environment or object they are approved for. As expected, the approval that leads to unhealthy narcissism comes from addictive objects and individuals in similar situations. It is already a condition that addictions are generally accepted to be caused by the inferiority complex [72].

According to Erik Erikson’s psychosocial development theory, everyone has responsibilities to fulfill and crises to overcome in certain life stages. Individuals who cannot overcome the burdens and crises at one stage go to the next stage with psychological disorders. For example, the most critical responsibility of a school-age child is their academic success, and the most important crisis is the teacher’s approval. The personality development of a child with poor academic success is disrupted because individuals cannot get approval from their teacher and, therefore, their environment. That is why individuals go through to adolescence as self-blaming individuals. However, if individuals fail to achieve academic success and turn to arts and sports accepted around them, they can get the approval they need [73]. As Adler said, an individual gets rid of the inferiority complex, realizes the compensation process, and reaches a healthy narcissism level [72].

This compensatory mechanism occurs through gaming in the age of play. In other words, children who do not receive acceptance from their parents or teachers might gain praise from their peers and compensate for their narcissism through their performance in a game, depending on their abilities [22]. The development of fine motor skills is achieved by increasing empathy and participating in the socialization process. In this way, the healthy narcissism and happiness that children need to meet the expectations of their environment are fulfilled.

Due to the narcissism scale subdimension problem, it was assumed that whether narcissism level is healthy or unhealthy depends on the participants’ happiness or gaming disorder level. Accordingly, the following hypotheses were established:

Responsibilities increase as age and class level increase [22]. In this case, while gaming disorder and happiness levels vary depending on age, class, and school, according to compensation theory, narcissism level is expected to remain constant in children who cannot cope with school period crises and therefore cannot get the approval they need from their environment [73].

**H_1.__1_.**  *Gaming disorder level positively associated with age and class.*

**H_1.__2_.**  *Narcissism level is not associated with age and class.*

**H_1.__3_.**  *Happiness level is negatively associated with age and class.*

As the socioeconomic situation improves, families are more likely to offer spaces for their children to express themselves [74]. In that sense, gaming disorder level and happiness vary in children who can conduct themselves in the areas such as academia, arts, games, and sports depending on their family income compared to the children who cannot do so, while the narcissism level is expected to remain constant according to the compensation theory [73].

**H_2.__1_.**  *Gaming disorder level is negatively associated with family income.*

**H_2.__2_.**  *Narcissism level is not associated with family income.*

**H_2.__3_.**  *Happiness level is positively associated with family income.*

Sibling ownership plays an important role in the development of narcissistic personality [22]. Although it is thought that the number of siblings will prevent gaming disorder due to increased socialization, rivalry between siblings may lead to the development of unhealthy narcissism in children with siblings, as in children without siblings [75]. So, while the levels of gaming disorder and happiness in children with siblings differ from those without siblings, the level of narcissism is expected to remain constant according to the compensatory theory [76].

**H_3.__1_.**  *Gaming disorder level is positively associated with the number of siblings.*

**H_3.__2_.**  *Narcissism level is not associated with the number of siblings.*

**H_3.__3_.**  *Happiness level is negatively associated with the number of siblings.*

Internet cafes and game consoles are an ideal area for children who cannot reach healthy levels of narcissism [77], as they offer the opportunity to meet with children who are like them [78]. It is expected that the levels of gaming disorder and happiness will vary, and the level of narcissism will remain constant according to the compensation theory depending on the frequency of going to internet cafes or game console halls [79].

**H_4.__1_.**  *Gaming disorder level is positively associated with the frequency of going to internet cafes or game console halls.*

**H_4.__2_.**  *Narcissism level is not associated with the frequency of going to internet cafes or game console halls.*

**H_4.__3_.**  *Happiness level is negatively associated with the frequency of going to internet cafes or game console halls.*

Children in need of attention spend time watching games when they cannot play. In this way, they want to overcome their lack of interest [80]. Children want to improve themselves and get rid of the inferiority complex by watching good players [81]. Depending on watching digital game videos, it is expected that gaming disorder and happiness will vary. The level of narcissism will remain constant according to the compensation theory.

**H_5.__1_.**  *Gaming disorder level is positively related to watching digital game videos.*

**H_5.__2_.**  *Narcissism level is not related to watching digital game videos.*

**H_5.__3_.**  *Happiness level is negatively related to watching digital game videos.*

Sharing digital game recordings on social media provides an opportunity for children who are trying to meet the need for approval to show their talents [82,83]. In that sense, it is expected that gaming disorder and happiness levels will vary depending on the recording and sharing of digital game videos on online platforms while narcissism level is expected to remain constant depending on the level of addiction, according to the compensation theory [84].

**H_6.__1_.**  *Gaming disorder level is positively related to the sharing of digital game videos on online platforms.*

**H_6.__2_.**  *Narcissism level is not related to the sharing of digital game videos on online platforms.*

**H_6.__3_.**  *Happiness level is negatively related to the sharing of digital game videos on online platforms.*

Gaming disorder is generally associated with the compensation of inferiority complex, and the primary purpose is to maintain the narcissism level [85]. Therefore, the gaming disorder level is expected to be related to unhealthy narcissism.

**H_7_.**  *Narcissism level is positively related to the gaming disorder level.*

Gaming disorder is a disorder caused by the lack of approval and interest from the social environment and many factors such as genetics and personality traits [86]. It is the effort of a child who cannot achieve long-term happiness to hold on to life with short-term pleasures [87]. Therefore, the gaming disorder level is expected to be negatively associated with happiness.

**H_8_.**  *Happiness level is negatively related to the gaming disorder level.*

Healthy narcissism is the admiration of the individual and being at peace with oneself [33]. It is difficult for a person with inner peace to be unhappy [88]. For this reason, it is expected that narcissism level and happiness level are positively associated.

**H_9_.**  *Happiness level is positively related to narcissism level.*

Unhealthy narcissism develops in children who cannot reach long-term happiness, pursue short-term pleasures, and take shelter in gaming disorder to get rid of the inferiority complex [89]. As a result, while healthy narcissistic individuals are happy, unhealthy narcissistic individuals are expected to be unhappy.

**H_10_.**  *Gaming disorder level has a mediating role between narcissism and happiness levels.*

The game played has an essential impact on gaming disorder. Many studies have shown that some games cause higher levels of gaming disorder [90]. Children with the highest gaming disorder level play the most LoL among games [91]. It was thought that LoL could be a moderator between narcissism and gaming disorder.

**H_11_.**  *Playing the LoL game has a moderating role between gaming disorder and narcissism.*

**Figure 1:**
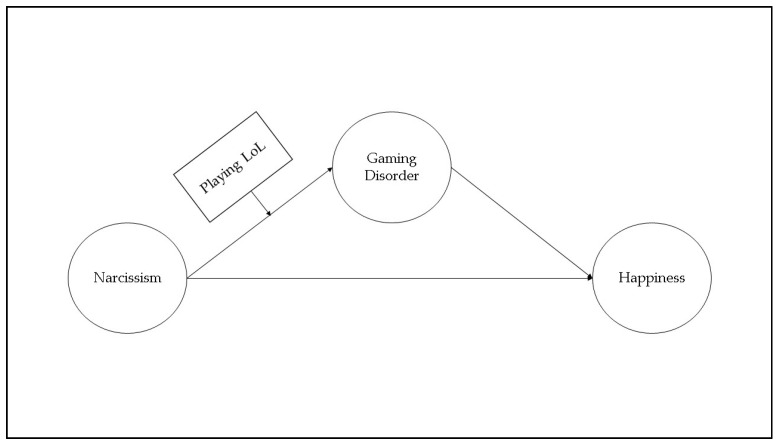
Conceptual diagram of research model (Model 7).

To test our hypotheses, direct, mediator, and moderator analyzes were performed as illustrated in Figure 1. First of all, direct, then mediator and moderator analyses were performed between the dependent, mediator, and independent variables. Age, school class, family income, and the number of siblings were used as control variables.

## 2. Materials and Methods

### 2.1. Study Design, Participants, and Procedure

Nesting qualitative data in quantitative design, one of the mixed research designs, was used in this study, and data were collected by two different methods:

1. In the quantitative cross-sectional research design, the convenience sampling technique was used in the descriptive model, and data were collected using the questionnaire method [92,93].

2. In the qualitative phenomenological research design, the descriptive sampling technique was used, and data were collected using the document analysis method [93].

The study was conducted in primary and secondary schools in the Bayrampaşa district, considering that it is similar to Turkey’s general socioeconomic and cultural texture. Permission to research in schools in the Bayrampaşa district was obtained from the Governorship of Istanbul and the Provincial Directorate of National Education.

The sample consists of 461 boys between the ages of 9 and 15 who attend 4th, 5th, 6th, and 7th classes in public schools in Bayrampaşa, where the study was conducted. Since many studies show that gaming disorder is more common in boys [94], girls were not included in the study to reach individuals with a high level of gaming disorder under limited time and economic conditions. The universe, which was estimated to be approximately 5,000,000 boys, attended public school in Turkey in the 4th, 5th, 6th, and 7th classes. With a 5% margin of error and a 95% confidence interval, 384 people can represent a group over 5,000,000 people [92]. That is why the sample represents the 9–15 age group of the population in Turkey.

### 2.2. Data Analysis

The data were collected in public schools in the Bayrampaşa district in May 2019. First of all, the informed volunteer parent form was delivered to parents through the parents’ associations. Subsequently, the signed consent forms were collected, the questionnaire was distributed to the children, who brought the signed document under the control of the school administrations, and data were collected. All collected data were analyzed with SPSS v24 and PROCESS v3.5 plug-in program. The Draw-a-Person test was interpreted qualitatively.

To see the suitability of the data for parametric analysis, the multiple normality test was carried out. It was observed that the skewness and kurtosis values of each of the three scales used in the study were within the minimum value range (±1.96) required for the multiple normality distribution [95]. It can be said that the data set structure was suitable for parametric analysis of multiple normal distribution values.

Factor analysis test was conducted with the help of IBM SPSS v24 to see the scale validity. The data set was rotated for all scales using the direct oblimin method. It was seen that only the 6th item of the Positive Subjective Well-Being dimension of the School Children’s Happiness Inventory did not have sufficient factor loading. The 6th item was removed from the list and analyzed again. The findings obtained showed that the total variance, KMO, and Bartlett’s test values explained for all scales were compatible with the studies where the scale validity was provided, and validity was accepted. When the scale’s reliability was examined, it was seen that Cronbach’s alpha values were 0.788 for DGAS, 0.708 for CNS, and 0.903 for SCHI. The scales have sufficient reliability values [95].

### 2.3. Measures

Digital Game Addiction Scale (DGAS) was used to determine the gaming disorder level of the students. The scale, adapted to Turkish by Yalçın Irmak and Erdoğan [96], was developed by Lemmens et al. [97]. Validity and reliability values are in the range of acceptable values for both the original scale and the adaptation study. We used the five-point Likert-type scale, which is one-dimensional.

Childhood Narcissism Scale (CNS) was used to determine the narcissism level of children. The one-dimensional scale was developed by Thomaes et al. [70] and was adapted to Turkish by Akın et al. [98]. The four-point Likert-type scale has ten items and has no items that need reverse coding. The scale measures the level of the narcissistic personality, not the level of unhealthy narcissism.

School Children’s Happiness Inventory (SCHI) was used to measure the long-term happiness level of children. The inventory was developed by Ivens [99]; the Turkish validity and reliability study was conducted by Telef [100]. The four-point Likert-type scale has a 30-item inventory and has two subdimensions expressing positive and negative subjective well-being and 15 items that need to be reverse coded.

The “Goodenough–Harris Draw-a-Person test” was used to better understand children’s inner world and provide quantitative data. Draw-a-Person test was first discovered by Florence Goodenough [101] and later developed by Dale B. Harris [102]. In the test with a pencil and blank paper, each figure drawn by the children is scored. It is assumed that the level of intelligence increases in direct proportion to the score increase. However, the Draw-a-Person test, which is also accepted as a projective test by clinical psychologists, gives information about children’s personality traits and spiritual world by interpreting the drawn figures. The Draw-a-Person test was used as a projective test and was interpreted qualitatively in the research.

## 3. Results

### 3.1. Descriptive Analysis

Descriptive statistics are shown in Table 1. The most participation was from the 12-year-old group. The 15-year-old group had the least participation. While the youngest age group was 9, the oldest age group was 15. The average age was 11.51; the standard deviation was calculated as 1.226. Of the participants, 277 of the children stated that their family income was low, and 184 stated that their family income was high. Most of the participants (57.2%) have at least one sibling. The most participation was from 7th-class children. The least participation was from 5th-class children. Most of the participants do not go to internet cafes or game console halls to play games; however, it was found that almost a quarter preferred these venues for games. It was seen that the most liked and played game by the children participating in the study was the PlayerUnknown’s Battlegrounds (PUBG) game. It was found that most children (78.3%) watched the videos of these games on social media or online video sites when they could not play the games they liked or to see the performances of better players. In the study, it was seen that almost one-third of the participants gave a positive answer to the question asked to learn whether they recorded the games they played and shared them on any platform.

Means and standard deviations of scales are shown in Table 2. The gaming disorder scale average (M = 2.0, SD = 1.5) is higher than the total score average (M = 1.2, SD = 0.8) of the participants on this scale. The narcissism scale average (M = 1.5, SD = 1.2) is similar to the average of the participants (M = 1.5, SD = 0.5) on this scale. The total score average of the participants in the happiness scale (M = 3.1, SD = 0.5) is higher than the scale’s original average (M = 2.5, SD = 1.5). It was observed that the gaming disorder level of the participants in the sample was lower than the general average, the level of narcissism was at the general average level, and the level of happiness was higher than the general average. It is meaningful that a participant group with a low level of gaming disorder and average narcissism has a high level of happiness.

### 3.2. Correlations

Correlations between variables are shown in Table 3. According to the table, while gaming disorder level increases with age and class, happiness decreases, and narcissism has no association (r = 0.191; r = −0.145, *p* < 0.01; r = −0.001, respectively, *p* > 0.05) (H_1.__1_, H_1.__2_, and H_1.__3_ were accepted). As family income increases, gaming disorder level decreases and narcissism and happiness increase (r = −0.092, *p* < 0.05; r = 0.122; r = −0.157, respectively, *p* < 0.01) (H_2.__2_ was not accepted; H_2.__1_ and H_2.__3_ were accepted). A significant negative relationship has been found between happiness and number of siblings, while there were no correlations between the number of siblings, gaming disorder level, and narcissism (r = −0.114, *p* < 0.05; r = −0.049; r = −0.036, respectively, *p* > 0.05) (H_3.__2_ and H_3.__3_ were accepted; H_3.__1_ was not accepted). There were associations between the frequency of going to internet cafes or game console halls and gaming disorder level and happiness, whereas there was no relationship with narcissism (r = 0.252; r = −0.202, *p* < 0.01; r = −0.025, respectively, *p* > 0.05) (H_4.__1_, H_4.__2_, and H_4.__3_ were accepted). There was a correlation between watching digital game videos and gaming disorder level and happiness, whereas there was no correlation with narcissism (r = 0.296; r = −0.138, *p* < 0.01; r = 0.061, respectively, *p* > 0.05) (H_5.__1_, H_5.__2_, and H_5.__3_ were accepted). The sharing digital game videos variable was positively related to gaming disorder level and narcissism, whereas it was negatively related to happiness (r = 0.196, r = −0.121, r = 0.116, respectively, *p* < 0.01) (H_6.__2_ was not accepted; H_6.__1_ and H_6__1_ were accepted). Gaming disorder level and happiness were related positively with narcissism (r = 0.108, r = 0.097, respectively, *p* < 0.05) while gaming disorder level was negatively correlated with happiness (r = −0.457, *p* < 0.01) (H_7_, H_8_, and H_9_ were accepted).

### 3.3. Hypothesis Testing

#### 3.3.1. Regression Analysis

To test our hypotheses, direct effect analyses were made between the independent and mediator and dependent variables, as seen in Table 4. Demographic data of the participants were used as control variables in these analyses. First of all, in Model 1, the effect of independent variable narcissism on mediator variable gaming disorder level was tested, and a significant positive impact was found (B = 0.14, *p* < 0.05, CI [0.0062, 0.2751]). Moreover, in Model 1, the effects of those who watch digital game videos and go to the internet cafe to play games on gaming disorder level were found to be positive (B = 0.50, B = 0.24, *p* < 0.001, CI [0.3120, 0.6822; 0.1367, 0.3334], respectively). H_4_ and H_5_ were accepted. The effects of narcissism and gaming disorder on happiness were tested in Model 2, and both had a significant impact (B = 0.13, B = −0. 25, *p* < 0.01, *p* < 0.001, CI [0.0508, 0.2050; −0.3036, −0.1980], respectively). H_8_ and H_9_ were accepted. In Model 2, it was found that family income increases happiness, and the number of siblings decreases happiness (B = 0.09, B = −0.09, *p* < 0.05, *p* < 0.01, CI [0.0040, 0.1684; −0.1557, −0.0301], respectively). H_2.__3_ and H_3.__3_ were accepted. To test the moderation analysis of those who play the LoL game, the interaction variable (Narcissism X LoL), which consists of narcissism and the variable representing those who play the LoL game, was computed. The effect of the interaction variable on gaming disorder level was tested, and results are shown in Model 3. It was understood that the moderation effect of those who played the LoL game was significant in the impact of narcissism on gaming disorder level (B = 0.81, *p* < 0.01, 95% CI [0.2259, 1.4055]).

#### 3.3.2. Mediation Analysis

Firstly, to perform the mediation analysis, the effect of the independent variable narcissism on gaming disorder level was tested and found to be a significant impact. Then, the impact of narcissism on both gaming disorder level and happiness was tested, and a meaningful impact was seen. Therefore, gaming disorder level was found to have a mediating effect in the effect of narcissism on happiness (γ = −0.0353, SE = 0.0176, 95% CI [−0.0724, −0.0037]), as shown in Table 5. Since the impact of narcissism on happiness was also significant (γ = 0.1279, SE = 0.0392, 95% CI [0.0508, 0.2050]), it was understood that the mediation effect was partial. According to these results hypothesis, H_10_ was accepted. 

#### 3.3.3. Moderation Analysis

**Figure 2:**
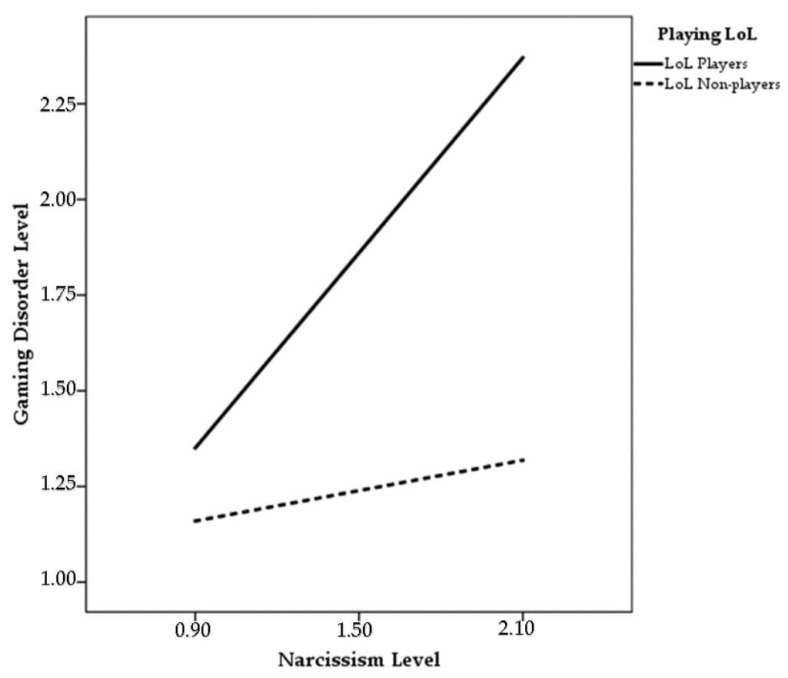
Moderation of Playing LoL on gaming disorder and narcissism.

The effect of the interaction variable (Narcissism X LoL) on gaming disorder level is shown in Table 4 and Model 3. According to these data, the impact of the interaction variable on gaming disorder level was found to be significant (B = 0.81, *p* < 0.01, 95% CI [0.2259, 1.4055]). Accordingly, it was understood that the effect of narcissism on gaming disorder level in LoL players increases much more than those who do not play LoL, as shown in Figure 2. According to these results, hypothesis H_1__1_ was accepted.

#### 3.3.4. Draw-a-Person Test Analysis

**Figure 3:**
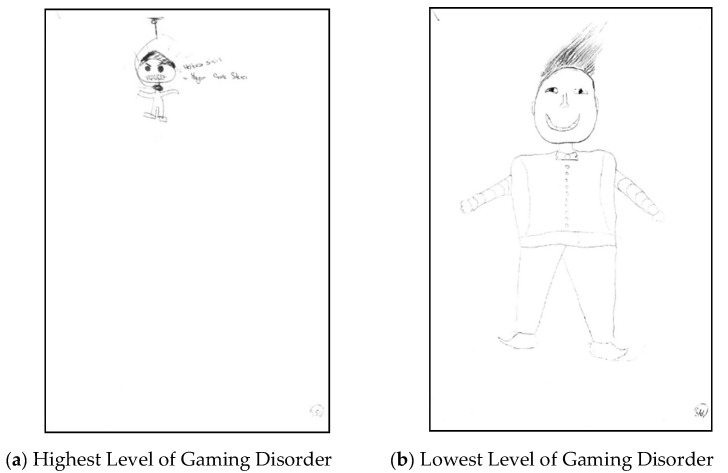
These pictures show the Draw-a-Person test drawings of two children with the highest and lowest scores on the DGAS. (**a**) The child who drew the picture is 13 years old. He scored 25 points from the DGAS (max. 28–min. 0), 19 points on CNS (max. 30–min. 0), and 33 points (max. 120–min. 30) on SCHI. (**b**) The child who drew the picture is 12 years old. He scored 0 points on the DGAS, 30 points on CNS, and 119 points on SCHI.

(a) Highest Level of GD: The words “Everybody is angry” and “Life is boring” were written on the picture, and the scenes of suicide show that mental health is deteriorating in a pathological dimension. Drawing the whole body on a very small area of the paper signals a lack of self-confidence and an inferiority complex. The body that faces left represents a dependent structure. Body lines are sharp and hard. It presents an aggressive personality trait. The size of the head compared to the body and the coloring of hair black are related to the inferiority complex [103]. Spooky mouth expresses insult, furrowed eyebrows express anger and unhappiness. The width of its jaw signals the need for approval. It can be concluded that social relations are impaired due to the absence of ears [103]. The neck is thin and long. It can be said that the impulse control mechanism is strong. His shoes show that he is economically free. In the survey, he stated that the family income is good, consistent with the picture. His arms are open, but they have no hands or fingers. It was understood that he is open to communication but has no friends, as shown in Figure 3a.
(b) Lowest Level of GD: The positive impression at first glance indicates that his mental health is good. Drawing the whole body in the middle and almost the entire area of the paper indicates a high level of self-confidence. The fact that his feet are facing both sides and the body is in the center indicates that he was interested in the future and the past. Body lines are light and oval. It indicates a soft and fragile personality trait. The normal size of the head relative to the body and the shape of the hair are associated with healthy narcissism [103]. The upward curved mouth expresses happiness. The width of its jaw signals the need for approval. It can be concluded that social relations are healthy from the normal size of his ears [103]. The neck is of normal length and width. It can be said that the impulse control mechanism is healthy. His shoes show that he can stand on his own feet and control his own life. In the questionnaire, he stated that the family income is medium, consistent with the picture. His arms are open, his hands are drawn, and his fingers are in normal condition. It was understood that he is open to communication and follows the rules, as shown in Figure 3b [103].

As a result, it was seen in the pictures how the gaming disorder level increases the levels of unhealthy narcissism and unhappiness. Therefore, it can be said that hypotheses H_7_, H_8_, H_9_, and H_10_ were accepted.

## 4. Discussion

When examined conceptually, we understood that playing digital games, narcissism, and happiness have both positive and negative aspects. The level of healthy narcissism increases as we move away from the inferiority complex and lack of interest and approval [22]. An individual who has reached a healthy level of narcissism either has never needed compensation or has gone through this process in a healthy way [22]. In that case, children do not need to suppress their unhappiness with momentary pleasures and try to satisfy their need for attention and approval from the digital world. They play digital games for personal growth and flow experience, not for compensation [23,39]. Thus, the quality of life and life satisfaction increase more and reach long-term happiness [38]. Individuals who cannot get rid of their inferiority complex and cannot meet their need for attention and approval play digital games to compensate for the narcissism and suppress their unhappiness with momentary pleasures [104]. Unless necessary interventions are made, unhealthy narcissism, gaming disorder, and unhappiness levels increase in relation to each other, and this turns into a vicious circle.

It was found that gaming disorder level is positively associated with age and class. When the literature was reviewed, found similar results were found [1,105,106,107,108,109]. As the age and class level increase, children’s awareness of life and possible problems will also increase. In this sense, as age increases, children’s gaming disorder levels will increase as an escape from problems. According to Erikson, every age period has certain responsibilities and crises. If these responsibilities are not fulfilled and crises are not overcome, the need for approval cannot be met [110]. Unless the need for approval is truly compensated, the individual cannot connect with real life and begins to diverge. The individual now turns to objects of short-term pleasure for which they are approved. As the compensation process is delayed, it becomes more difficult for the individual to return to real life, and gaming disorder increases [111].

Although gaming disorder level was significantly related to family income, when the literature is examined, it is seen that similar results are obtained [108,109,112,113]. Therefore, it can be said that the gaming disorder level varies negatively depending on family income. For the compensation process to occur, the individuals must be aware of their abilities. These skills must then be demonstrated and developed over time to gain the approval of the environment. Awareness, demonstration, and development of these abilities often depend on the well-being of the socioeconomic situation [74]. Children who are unaware of their abilities and who do not have the opportunity to show and develop them cannot meet the need for approval. Failure to meet the need for consent is the most crucial factor driving the individual into gaming disorder [104].

It was found that gaming disorder level is not associated with the number of siblings. However, when the difference in scale average points is examined, it is seen that it is similar to the literature [112,113]. Positive and strong relationships in the family play an important role in satisfying the individual’s need for approval. Especially as siblings spend more time with each other, they can show each other their abilities more quickly and meet the need for approval more easily. So, as the number of siblings increases, the individual’s connection with real life is supposed to get stronger, and dependence decreases. However, Adler’s theory predicts that as the number of siblings increases, happiness decreases. Interestingly, although gaming disorder level is low, we also found that happiness is inversely proportional to the number of siblings. It has been observed that factors affecting sibling relationships, such as birth order and competition, mentioned by Adler [22], affect children’s happiness as much as gaming disorder level.

It was found that gaming disorder level was positively related to the frequency of going to internet cafes and game console halls. No similar study was found in the literature. However, it was seen that the frequency of going to internet cafes and game console halls and the duration of use were related to the results of these studies [108,109,112,113]. Children who cannot complete the compensation process try to meet their approval needs in virtual environments that they can access at home. However, this is sometimes not possible due to parents’ blocking and sometimes lack of access. For this reason, children cannot find an ideal place to satisfy their approval need other than internet cafes and game console halls. The need for consent is met more when they see children in the same situation [79]. Therefore, the direct relationship between the frequency of going to these places and the level of gaming disorder cannot be denied.

It was found that gaming disorder level was positively correlated with watching digital game videos. No similar study was found in the literature. It was stated that watching digital game videos partially carries digital game functions such as developing skills, social communication, and entertainment [114]. The fact that children have limited access to the internet and some cannot complete the compensation process pushes them away from the environment they obtain their consent from. Children who experience dissatisfaction in real life watch videos of digital games they play as an alternative to return to the environment they have received their approval from as soon as possible. They break away from real life and learn new techniques from good players in videos, improving their skills in the virtual world. Thus, when they get the opportunity to play, they greatly eliminate the need for approval, as they can demonstrate more skills. As a result, it can be said that digital game videos increase gaming disorder [80].

It was found that gaming disorder level was positively related to sharing digital game videos on online video platforms. No similar study was found in the literature. However, it was observed that the digital games with the most shared videos are also the games that cause the most gaming disorder [115]. Therefore, it can be said that the findings were significant and theoretically supported. Although the need for approval is provided temporarily from digital games, the approval of real people is always more satisfying. For this reason, children want to show their talents in the virtual world to real people on digital platforms [84]. As the approval from these platforms rises, the dependence on both digital games and the virtual world increases.

It was found that narcissism level was positively correlated with the gaming disorder level. When the literature was examined, it was seen that similar results were obtained [53,116,117]. In the individual’s relationship with the self and the object, unhealthy narcissism expresses the return of attention to self. In contrast, the inferiority complex refers to the focus of attention on the object. As the addiction, which is an effort to get rid of the inferiority complex, increases, the self is approached [85]. Therefore, it can be said that as gaming disorder increases, unhealthy narcissism also increases.

It was found that happiness level was negatively associated with the gaming disorder level. When the literature was examined, it was seen that similar results were obtained [54,55,56,59,61,62,63,64,65,66]. Happiness is associated with meeting an individual’s need for approval in life. The approval needed is sometimes long-term and sometimes short-term. For example, graduating from a university earns long-term approval from the community, while a high score from a game earns short-term approval. It is impossible for individuals who depend on short-term approvals to be happy for a long time by failing to meet the long-term approval requirement [87].

It was found that happiness level was positively correlated with narcissism level. Although some studies in the literature suggest otherwise [48,52], most studies support the findings [41,42,43,44,45,46,47,48,49]. Because of the inconsistency of the scales of narcissism, it was normal to have opposite results in this way [71]. Healthy narcissism refers to individuals’ balanced relationships with themselves and their environment. The achievement of this relationship to the desired level depends on the approval of individuals from their environment [88]. Individuals achieve the approval they need by fulfilling what is expected of them, and thus their healthy narcissism and happiness levels increase.

It was found that gaming disorder level had mediating role between narcissism and happiness levels. No similar study was found in the literature. However, compensation and psychosocial development theories supported gaming disorder level prediction as a mediator variable of narcissism and happiness [22,73]. We also found the moderation effect of LoL in the impact of narcissism on gaming disorder level. As a result, it was discovered that the impact of narcissism on gaming disorder level in LoL players is much greater than in non-LoL players [90].

The most notable component of this study is the combination of qualitative and quantitative methodologies. In this sense, the Draw-a-Person test was used to supplement quantitative analyses with qualitative data. As a result of the analysis, we observed that the quantitative findings were consistent with the qualitative results of the Draw-a-Person test. Both a qualitative verification of the scales and a quantitative interpretation of the Draw-a-Person test were made. Karen Machover was the first to state that it was possible to understand children’s personalities with a draw-a-human test [118]. Many studies confirmed this technique [119,120,121]. Significant findings were obtained, especially in the tests conducted for addicted individuals [122,123].

## 5. Conclusions

Findings in the research were in line with previous studies. However, it was the first study to examine gaming disorder level within the framework of compensation theory and show that it acts as a mediating between narcissism and happiness. At the same time, it showed the consistency of both the scales and the Draw-a-Person test by taking the findings from the Draw-a-Person test together with the information on the scales.

In the findings, while gaming disorder and happiness levels were associated with demographic data, the level of narcissism was not. It was observed that the compensation theory could explain the findings. If so, it will be efficient to make the necessary interventions for gaming disorder level within the framework of compensation theory.

However, this compensation process does not seem possible today, where the traditional family structure is disrupted, the neighborhood culture is lost, the people become lonely as they get crowded, the young population decreases gradually, and the streets become insecure [124]. As a solution, digital game platforms as a silencing pacifier are offered so that children abandoned in virtual environments as dangerous as outside do not cry out of indifference and unacceptance.

Scientific studies have proved that digital games have many educational and instructive benefits besides entertainment [64]. These findings show that digital games have several functions that traditional games have. However, the addictive feature of digital games does not appear in conventional games. This situation reveals the problem of abuse, which is one of the concepts related to addiction. Abuse is generally associated with addictive substances and refers to a problem originating from the individual [125]. However, the abuse here is done by society, not by the individual. How can society abuse digital games? Due to the concrete construction in big cities, children cannot find a place to play for themselves [126]. Neighborhood culture is not formed due to irregular economic or social migrations [127]. The birth rate decreases with the increase of the education level, and the number of parents who think that raising children consists of meeting the child’s financial needs is increasing [128]. Instead of health professionals, those who overcome all these problems are digital game producers, which have one of the largest markets in the world and are thought to contribute to humanity with their new games every day. In this system, digital game producers make parents, who are their real “customers”, happy up to a point. However, by pushing children, who are their main “users”, to addiction, they drag them into an unhealthy narcissism and make them unhappy.

So, for gaming disorder, macro-level interventions should be done primarily instead of micro-interventions. For this reason, it would be appropriate to put into practice macro models that will be prepared within the framework of system theory instead of the currently used medical model [129].

## 6. Assumptions, Limitations, and Suggestions for Future Studies

It is assumed that the children participating in the research answered the questions honestly in line with their own free will and without being under the influence of anyone. The narcissism scale does not have subdimensions. It was assumed that whether the level of narcissism is healthy or unhealthy can be determined by looking at the levels of happiness and gaming disorder.

The sample of the study is limited to 461 participants, consisting of 4th-, 5th-, 6th-, and 7th-class students studying in the primary and secondary schools affiliated with Istanbul Bayrampaşa District National Education Directorate in the 2018–2019 academic year. Considering the economic dimension of the study, it was assumed that the level of gaming disorder was higher in boys, so girls were not included in the study. Therefore, the study is limited to male participants. All the information and findings in the research are limited to those obtained from the demographic form, scales, and Draw-a-Person test.

Since the Childhood Narcissism Scale does not have subdimensions, it was not understood which type of narcissism this scale measures. In today’s world, where narcissism is spreading rapidly, preparing a more qualified scale will be beneficial for researchers. It is necessary to research different genders, socioeconomic groups, and adults to reach more understandable results.

## Figures and Tables

**Table 1:** Descriptive statistics.

Variable		f	%	M	SD
Age	9–11	351	2.6	0.40	0.490
	12–15	110	24.1		
School Class	4th	126	27.3	5.61	1.196
	5th	75	16.3		
	6th	112	24.3		
	7th	148	32.1		
Family Income	Low	277	60.1	0.40	0.490
	High	184	39.9		
Number of Siblings	No	197	13.2	1.30	0.688
	One	203	44.0		
	More than one	61	42.7		
Frequency of Going to Internet Cafe or Console Halls	Never	332	72.0	0.40	0.692
	Once a Week	74	16.1		
	A few times a week	55	11.9		
Watching Digital Game Videos	Yes	361	78.3	0.78	0.413
	No	100	21.7		
Sharing Digital Game Videos	Yes	146	31.7	0.32	0.466
	No	315	68.3		
Total		461	100		

**Table 2:** Means and standard deviations of measures.

Measure	Min	Max	Scale		Sample	
			M	SD	M	SD
Digital Game Addiction	0 (Never)	4 (Always)	2.0	1.5	1.2	0.8
Childhood Narcissism	0 (Not at all true)	3 (Completely true)	1.5	1.2	1.5	0.5
School Children’s Happiness	1 (I disagree a lot)	4 (I agree a lot)	2.5	1.5	3.1	0.5

**Table 3:** Correlations.

		1	2	3	4	5	6	7	8	9	10
1	Age	1									
2	Income	−0.127 **	1								
3	Number of Siblings	0.053	−0.07	1							
4	School Class	0.881 **	−0.139 **	0.087	1						
5	Sharing DGV	−0.041	−0.048	0	−0.032	1					
6	Frequency of GICCH	0.136 **	−0.015	0.155 **	0.093 *	0.088	1				
7	Watching DGV	0.005	−0.031	−0.111 *	−0.008	0.223 **	0.100 *	1			
8	Playing LoL	0.256 **	−0.107 *	−0.011	0.240 **	0.009	0.036	0.032	1		
9	Narcissism	−0.001	0.122 **	−0.036	−0.015	0.121 **	−0.025	0.061	0.015	1	
10	Gaming Disorder	0.191 **	−0.092 *	−0.049	0.132 **	0.196 **	0.252 **	0.296 **	0.185 **	0.108 *	1
11	Happiness	−0.145 **	0.157 **	−0.114 *	−0.09	−0.116 *	−0.202 **	−0.138 **	−0.138 **	0.097 *	−0.457 **

Notes. DGV = digital game videos, LoL = League of Legends, GICCH = going to internet cafes or console halls ** *p* < 0.01, * *p* < 0.05., Bold values = Correlation values between variables are statistically significant.

**Table 4:** Main effects on dependent variables.

Variable	Model 1: Gaming Disorder			Model 2: Happiness			Model 3 (Moderation): GD		
	B	SE	*p*	B	SE	*p*	B	SE	*p*
(Constant)	−0.77	0.50	0.126	3.69	0.29	0.000	−0.59	0.49	0.231
Age	0.18	0.07	0.008	−0.06	0.04	0.093	0.18	0.06	0.006
Family Income	−0.11	0.07	0.138	0.09	0.04	0.040	−0.11	0.07	0.145
Number of Siblings	−0.07	0.06	0.185	−0.09	0.03	0.004	−0.07	0.06	0.215
School Class	−0.09	0.07	0.158	0.06	0.04	0.119	−0.10	0.07	0.126
Sharing DGV	0.23	0.08	0.007	−0.04	0.05	0.373	0.24	0.08	0.004
Frequency of GICCH	0.24	0.05	<0.001	−0.04	0.03	0.174	0.24	0.05	0.000
Watching DGV	0.50	0.09	<0.001	−0.02	0.06	0.746	0.49	0.09	0.000
Playing LoL	0.47	0.16	0.003	−0.10	0.09	0.284	0.45	0.16	0.005
Narcissism	0.14	0.07	0.040	0.13	0.04	0.001	0.10	0.07	0.168
Gaming Disorder				−0.25	0.03	0.000			
Narcissism X LoL							0.81	0.30	0.007
F		13.60			16.43			13.15	
*p*		<0.001			<0.001			<0.001	
R2		0.214			0.267			0.226	

*Notes.* GD = gaming disorder, DGV = digital game videos, LoL = League of Legends, GICCH = going to internet cafes or console halls.

**Table 5:** Direct and indirect effects.

Direct Effect of Narcissism on Happiness					Unstd.	SE	LLCI	ULCI
Narcissism	>	Happiness			0.1279	0.0392	0.0508 *	0.2050 *
Indirect Effect of Narcissism on Happiness								
**Independent**	**Mediator**			**Dependent**	**Unstd.**	**SE**	**LLCI**	**ULCI**
Narcissism	>	Gaming Disorder	>	Happiness	−0.0353	0.0176	−0.0724 *	−0.0037 *

* The indirect effect is significant if there is no 0 (Zero) between the LLCI and ULCI values.

## Data Availability

The data presented in this study are available on request from the corresponding author. The data are not publicly available because they are a part of a developing dataset that will be used in the future for different studies.
